# Expression of a chimerical pcDNA encoding influenza virus M2 protein and HSP70 gene in eukaryotic cell lines

**Published:** 2011-01-10

**Authors:** F Fotouhi, MT Kheiri, M Esghaei, B Heydarchi, B Farahmand, V Mazaheri

**Affiliations:** 1 Influenza unit, Department of Virology, Pasteur Institute of Iran, Tehran, Iran; 2 Department of Virology, Iran University of Medical Sciences, Tehran, Iran

## 

The critical obstacle in developing of an effective human influenza vaccine is continuous antigenic variation of influenza A viruses occurred each year. Conserved antigens are important candidates for vaccines because it is not necessary to match the designed strains with circulating strains. M2 as an ion channel protein induce protective immunity. On the other hand, HSP70 is a molecular chaperon and immunostimulatory component, capable of eliciting effective humoral and cellular immunity against infectious disease. Genetically fusing antigens to HSPs leads to the enrichment of DNA vaccine potency.

In the present study, a chimerical DNA plasmid carrying influenza virus M2 protein and HSP70 Gene was constructed and the protein expression in eukaryotic cell line was evaluated. This construct could be used as a potent DNA vaccine. To gain this aim, Influenza A/New Caledonia/20/99 (H1N1) was inoculated into MDCK cell line. The supernatant was collected after 18 hours and total RNA was extracted using Easy-red (iNtRON) solution. Complementary DNA synthesis was carried out by RevertAid First Strand cDNA synthesis kit using uni-12 primer. Full length M2 gene (300bp) was amplified by polymerase chain reactions using designed primers. The PCR product was run on 2% agarose gel following by purification of specific band, cloned into pGEM-T Easy cloning vector (promega) and completely sequenced. The M2 gene was digested from T-vector and subcloned into the pcDNA3.1. *Leishmania amazonesis* heat shock protein (HSP70) gene was obtained by digestion of pGEM II- HSP70 and subcloned into pCDNA3.1. The digested M2 gene was then subcloned into the N-terminal of HSP70 in pcDNA3.1. Recombinant plasmids were transfected into COS-7 cells to evaluate protein expression.

The presence of M2 and HSP70 genes were confirmed by PCR, restriction enzyme analysis and electrophoresis. All the constructs were then verified by DNA sequencing. The vaccine candidates were transfected into COS-7 cells and protein expression was confirmed by indirect Immunofluorescense test, ELISA and western blotting. In ongoing project, the Immunomodulatory effect of HSP70 construct on DNA vaccine efficacy in animal models will be evaluated.

